# Machine Learning-Assisted Surface-Enhanced Raman Spectroscopy Detection for Environmental Applications: A Review

**DOI:** 10.1021/acs.est.4c06737

**Published:** 2024-11-13

**Authors:** Sonali Srivastava, Wei Wang, Wei Zhou, Ming Jin, Peter J. Vikesland

**Affiliations:** † Department of Civil and Environmental Engineering, Virginia Tech, Blacksburg, Virginia 24061, United States; ‡ Virginia Tech Institute of Critical Technology and Applied Science (ICTAS) Sustainable Nanotechnology Center (VTSuN), Blacksburg, Virginia 24061, United States; § Department of Electrical and Computer Engineering, Virginia Tech, Blacksburg, Virginia 24061, United States

**Keywords:** Surface-Enhanced Raman Spectroscopy, Machine Learning, Environmental Pollutants

## Abstract

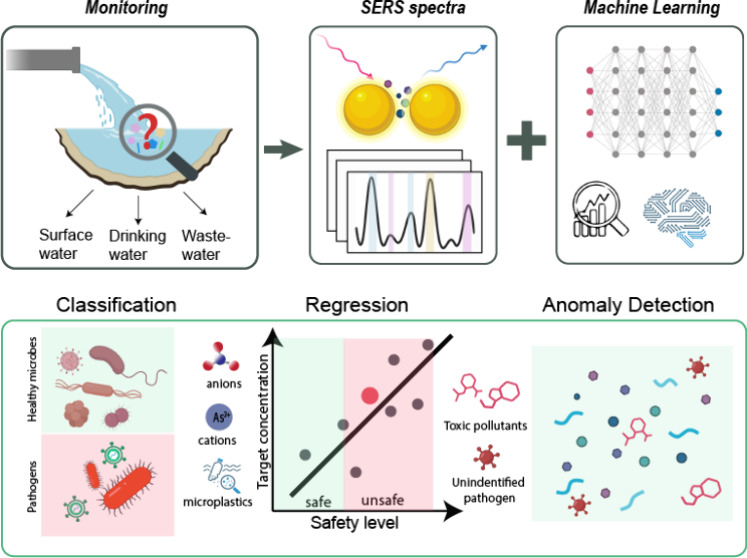

Surface-enhanced Raman spectroscopy (SERS) has gained significant attention for its ability to detect environmental contaminants with high sensitivity and specificity. The cost-effectiveness and potential portability of the technique further enhance its appeal for widespread application. However, challenges such as the management of voluminous quantities of high-dimensional data, its capacity to detect low-concentration targets in the presence of environmental interferents, and the navigation of the complex relationships arising from overlapping spectral peaks have emerged. In response, there is a growing trend toward the use of machine learning (ML) approaches that encompass multivariate tools for effective SERS data analysis. This comprehensive review delves into the detailed steps needed to be considered when applying ML techniques for SERS analysis. Additionally, we explored a range of environmental applications where different ML tools were integrated with SERS for the detection of pathogens and (in)organic pollutants in environmental samples. We sought to comprehend the intricate considerations and benefits associated with ML in these contexts. Additionally, the review explores the future potential of synergizing SERS with ML for real-world applications.

## 1 Introduction

Comprehensive environmental monitoring, spanning wastewater, drinking water, surface water, and air quality, has the potential for early detection of contaminants and pollutants.^1^ Increased integration of public health and environmental monitoring is essential for safeguarding health, preserving ecosystems, and upholding regulatory standards. Environmental engineers have diligently worked to detect contaminants, including pathogenic bacteria and viruses, microplastics, per- and polyfluorinated substances (PFAS), and heavy metals within environmental matrices.^2,3^ Detection and monitoring of these contaminants, whether in water, wastewater, or air, is crucial and aids in understanding contaminant fate and transport while offering substantial benefits, including community-level surveillance and the potential for effective epidemic management.^4,5^ To achieve rapid monitoring in environment matrices, there is a need for techniques that offer high sensitivity, cost-effectiveness, and field adaptability. Conventional gold standard methods for pathogen detection, such as enzyme-linked immunosorbent assay (ELISA) and polymerase chain reaction (PCR), or for (in)organic pollutants, such as chromatography-based and mass spectrometry (MS)-based techniques, have long been staples of environmental analysis.^6−8^ While these methods are recognized for their accuracy and sensitivity, they often come with high costs and are challenging to adapt to resource-limited settings.^9^ Additionally, diverse types of contaminants necessitate different detection techniques and often require specialized personnel and resource-intensive methods, thus limiting their universal application.

Surface-enhanced Raman spectroscopy (SERS) has emerged as an alternative approach with the capacity to detect a wide range of analytes from biological pathogens to emerging contaminants such as PFAS, and microplastics.^10,11^ SERS enhances the intrinsic Raman scattering of molecules that are associated with noble-metal nanoparticles or nanostructures.^12^ This enhancement facilitates the detection and identification of trace quantities of analytes at concentrations that can approach the single-molecule level.^13^ With rapid advances in nanofabrication and synthesis, SERS has gained increasing attention for environmental analysis.^14−16^ SERS stands out as a valuable tool for advancing environmental monitoring due to its capacity to offer cost-effective, highly sensitive, and adaptable approaches that are suitable under challenging environmental conditions.^17−19^

However, as the technology has evolved, SERS analysis has exhibited certain challenges, such as managing vast volumes of spectral data, addressing overlapping Raman peaks, and dealing with spectral artifacts.^20−22^ The acquisition speed and the information density contained with SERS spectra are notable. SERS measurements can rapidly accumulate substantial amounts of spectral data (ranging from megabytes to gigabytes) from a single sample within minutes and this can challenge data handling and limit comprehensive analysis. Traditionally, researchers have primarily focused on individual high-intensity SERS peaks, thereby constraining the comprehensive and impartial interpretation of the data.^23^ Moreover, the complexity of environmental matrices, replete with various impurities and molecules that may share similar or closely overlapped SERS peaks, presents challenges in distinguishing and interpreting the data.^19^ To tackle these complexities and make full use of the information obtained through SERS, researchers are turning to data analysis and machine learning (ML). While there have been several excellent review papers on the application of ML in SERS,^24−27^ there remains a significant gap in the literature specifically addressing the fusion of these techniques for environmental pollutant analysis. Despite substantial advancements in SERS and ML, integrating these fields as standardized tools for environmental monitoring requires further exploration and review.

To bridge this gap, we review recent studies that used SERS in combination with ML (SERS-ML) methods, including multivariate analysis within environmental settings. We begin by providing an overview of SERS and ML and outlining detailed steps to consider when employing ML for SERS analysis. We aim to offer a comprehensive and comparative analysis of different ML techniques applied to enhance the use of SERS for the detection of pathogens–bacteria, viruses, or their nucleic acids; organic pollutants–polyaromatic hydrocarbons (PAHs), organophosphorus pesticides (OPPs); and inorganic pollutants–anions and heavy metals and an emerging pollutant: microplastics. Finally, we outline prospective advancements for using SERS with ML in environmental settings. We discuss future scope, limitations, and key considerations for deploying SERS-based sensors in real-world applications.

## 2 Introduction to SERS

SERS is a powerful analytical technique for chemical and biochemical detection, with the potential for single-molecule sensitivity.^28^ SERS enhances the intrinsic Raman signal of target molecules by several orders of magnitude using metallic nanoparticles or nanostructures. The mechanisms responsible for SERS include electromagnetic field amplification and charge transfer processes and a detailed discussion can be found elsewhere.^29,30^ The combined effect of these enhancement mechanisms underscores SERS’ remarkable capability to detect molecules with ultrahigh sensitivity.^31,32^ SERS provides distinct molecular vibration fingerprinting patterns associated with molecular constituents, chemical bonds, and macromolecular configurations.^12^ As a molecule-specific approach for analyte detection, SERS enables the identification of diverse targets without requiring extensive sample pretreatment.

SERS approaches for molecule detection can be classified as label-free or labeled.^28^ In label-free SERS, the target molecule directly interacts with the SERS-active substrate and results in production of unique Raman spectra. Premasiri et al. demonstrated the advantages of SERS over bulk Raman in differentiating Gram-positive *Bacillus* strains and Gram-negative bacteria such as *Escherichia coli* and *Salmonella typhimurium*.^33^ Using a gold nanoparticle-covered SiO_2_ substrate, they achieved a ∼10^4^ enhancement factor, enabling species and strain distinction at the single-cell level. Additionally, SERS effectively minimizes fluorescence interference, a major issue in bulk Raman spectra, particularly for biological samples. The fluorescence quenching effect occurs due to the energy transfer from the excited chromophores to the metal surface that shortens the fluorescence lifetime, allowing the Raman signal to dominate and resulting in cleaner, more accurate spectral data.^34,35^ Numerous studies have explored label-free SERS detection of molecules, encompassing a broad range from biological entities to emerging pollutants such as PFAS, microplastics, illicit drugs, and more.^36−38^ However, the major limitation is multiple peaks overlapping in SERS spectra, which complicates the identification of specific molecules. Table S1 summarizes the peak assignments for various environmental contaminants. Since multiple groups often share overlapping Raman bands, distinguishing them for multianalyte identification can be challenging. Additionally, SERS enhancement mechanisms are highly distance- and orientation-dependent, with only molecular components within ∼10 nm of the substrate being preferentially enhanced.^32,39^ This spatial sensitivity leads to variability in SERS spectra across different substrates, further complicating standardization of the technique.

Although label-free detection has several advantages, it is also limited in its ability to identify molecules that exhibit weak or negligible Raman signals, such as viruses and heavy metals. In these instances, SERS tags have been developed by attaching intrinsically strong Raman scattering molecules (also known as Raman reporters) to the surfaces of nanoparticles, creating a distinct SERS spectrum of the Raman reporter.^40^ The incorporation of biorecognition elements, such as antibodies or aptamers, into the SERS tags enables them to bind specifically to targeted molecules. This is called labeled SERS and is particularly effective for biomolecular identification and quantification, including viruses and their antigens, pathogenic bacteria, and biomolecules.^41−43^

## 3 Decoding Machine Learning: Understanding the Essentials

ML is a subset of artificial intelligence that concentrates on developing algorithms and statistical models.^44^ These models empower computers to learn and enhance their performance based on data or experiences, thus enabling them to make predictions, classifications, and decisions.^45^ Within the domain of ML, we work with both training and test data sets. The training data set is instrumental in instructing the model, as algorithms generate a model that minimizes errors and provides an optimal fit.^44^ Subsequently, the model undergoes testing with test data to evaluate its accuracy. In this context, data sets can be classified into supervised and unsupervised learning. In supervised learning, algorithms learn to make predictions based on prelabeled data.^46^ These training data sets consist of input features along with their corresponding output features. The primary goal is for the algorithm to discern robust relationships between the input features and the target outputs, thereby enabling accurate predictions on new, unseen data. Prominent examples of supervised learning algorithms are regression, decision trees, and neural networks. In contrast, unsupervised learning represents an alternative approach within ML.^47^ Here, algorithms are designed to uncover patterns within unlabeled data. The training data comprises input features and lacks corresponding target outputs. The algorithm’s main goal is to unveil inherent patterns and relationships within the data, which can be leveraged to cluster similar data points together. An extensive overview of multivariate analysis techniques, ML, and deep learning methods, along with examples of their application in SERS is provided in Table 1.

**Table 1:** An Overview with Comparative Analysis of ML Tools—Including Unsupervised Learning, Supervised Learning, And Deep Learning Methods with Examples for Their Application to SERS Measurements

**Model**	**Category**	**Description**	**Examples of SERS with ML**	**Remarks**
**Multivariate analysis**				
**Principal Component Analysis (PCA)**(48)	Unsupervised	Dimension reduction method: Used to reduce the dimension of large data sets while preserving as much data variance as possible.	Sensing the illicit drug fentanyl in wastewater at 0.8 ppb concentration.^36^	Valuable technique for dimensionality reduction, noise reduction, and data visualization.
		Principal components (PC) are new variables constructed as a linear combination of the initial variables.	Multiplex SERS detection of PAHs,^50^ microplastics,^51^ pesticides,^52−54^ and polychlorinated phenols.^55^	Assumes that the underlying data relationships are linear.
		PCs explain the maximum amount of variance in the data.	Differentiation of pathogens: bacteria,^56−59^ viruses.^60^	Sensitive to outliers. Mostly works for continuous numerical data.
**Partial Least Square Regression (PLSR)**(61)	Supervised	Deals with data sets with multiple independent variables and one or more dependent variables.	Predicting concentration of the bacteriophage Phi6 using *Pseudomonas syringae*,^62^ viral loads of Hepatitis B serum.^63^	Effective when there is collinearity (high correlation) among the independent variables.
				Also, useful when independent variables are large compared to the number of observations.
		Performs dimensionality reduction by creating a new latent component that captures maximum covariance.		
**K-means clustering**(64)	Unsupervised	Primarily used for pattern recognition and clustering tasks.	Identification of methicillin-resistant and methicillin-sensitive bacteria;^59^ cancer cells;^65^ cancer biomarkers.^66^	Easy to implement and computationally efficient, suitable for large data sets.
		Operates on the principle that similar data points tend to be close to each other in a feature space.		Assumes that clusters have equal variance.
		Each data point is assigned to the majority class among its K nearest neighbors based on a chosen distance metric (e.g., Euclidean distance).		Sensitive to outliers.
		Sensitive to the choice of K and the distance metric.		Requires specifying the number of clusters (K) in advance.
**Hierarchical Clustering**(67)	Unsupervised	Aims to group data points into clusters based on their similarity.	Differentiation of *Bacillus* strains, often used with PCA.^56,57^	Provides a visual representation of clustering.
		Unlike other clustering methods, hierarchical clustering organizes data in a hierarchical tree-like structure called a dendrogram.		No need for predefined clusters.
		Can be either agglomerative (bottom-up), which starts with individual data points as clusters and merges them iteratively, or can be divisive (top-down), which starts with all data points in one cluster and splits them recursively.		Computationally intensive.
**Machine Learning (ML) models**				
**Random Forest (RF)**(68)	Supervised	Ensemble ML technique that combines multiple decision trees to make robust predictions.	Multiplex detection of antibiotic resistance genes;^69^ detection of PAHs^70^ in water	Robust to overfitting. Can handle linear and nonlinear data.
				For large data sets, RFs can be computationally intensive and may require tuning of hyperparameters.
		RF identifies the most influential feature by measuring its importance for prediction.		
		Can be used for both classification and regression.		
**Support Vector Machine (SVM)**(71)	Supervised	Works by finding an optimal hyperplane that best separates different classes in the data while maximizing the margin between them. A kernel function transforms input data into a higher-dimensional space, allowing SVM to find linear or nonlinear decision boundaries.	Detection of PAHs.^72^	Highly effective for both linear and nonlinear classification.
			Diagnosis of viral infection to bacterial growth;^73^ detections of different stains of *Escherichia*. *coli*;^74^ detections of SARS-CoV-2^75^	Regularization and margin maximization prevent overfitting. However, the choice of kernel and tuning parameters is crucial.
		Particularly effective in high-dimensional spaces and complex data distributions.		
		Ability to handle nonlinear relationships through kernel functions, thus making it versatile.		
**Logistic Regression (Logit model)**(76)	Supervised	Used for binary classification problems.	Classification of miRNAs;^77,78^ SARS-COV-2 variants.^79^	Straightforward and easy-to-implement algorithm.
				Provides interpretable results.
		Models the relationship between a binary outcome (0 or 1) and one or more independent variables by estimating probabilities.	Detection of microplastics.^80^	
				Assumes a linear relationship between the predictor variable and the log odds of the outcome.
		Uses the logistic function to transform a linear combination of input features into a probability score.		
**Deep learning algorithms**				
**Artificial Neural Network (ANNs)**(81)	Supervised	Class of ML models inspired by the human brain’s neural structure.	Monitoring cellular drug responses.^82^	ANNs can approximate complex functions, making them versatile for diverse tasks.
		Consists of layers of interconnected nodes, organized into input, hidden, and outer layers.	Industrial wastewater source tracing.^83^	Interpretability can be challenging due to the complex, nonlinear nature of ANNs.
		Key features of ANNs include their ability to learn complex, nonlinear relationships from data through the training process.	Single-molecule detection using SERS.^84^	Need large volumes of labeled data to perform well.
		Training involves adjusting the weights and biases of connections between neurons to minimize prediction errors.		
		Often requires substantial amounts of data and computational resources for training.		
**Convolutional Neural Network (CNN)**(85)	Supervised	Use convolutional layers to detect patterns in the input data. Convolution involves applying filters to small overlapping regions of the input.	Multiplex detection of PAHs,^86^ cancer detection,^87^*Salmonella* serotype identification.^88^	Automatically learns local features and spatial hierarchies.
		CNNs typically have one or more fully connected layers.		Requires large amounts of labeled training data.
		CNNs use weight sharing to reduce the number of parameters.		Computationally intensive.
**Multilayer Perceptron (MLP)**(89)	Supervised	MLP is a feedforward ANN.	Determination of *Mycobacterium tuberculosis* infection and drug resistance.^90^	Not all ANNs are MLP. The distinction lies in the specific architecture and connectivity patterns within the network.
		Information flows through the network in a forward direction.		
**Residual Network (ResNet)**(91)	Supervised	The core idea is to use residual blocks consisting of a shortcut connection (identity mapping) that bypasses one or more layers.	Identification of methicillin-resistant and -susceptible *Staphylococcus aureus*,^92^ multiplex detection of organophosphorus pesticides in environmental water.^93^	Introduced to address the challenge of training very deep neural networks.
				Effectively maintain previous gradient information by shortcut connection.

### 3.1 Building and Evaluating ML Models in SERS Analysis

**Figure 1:**
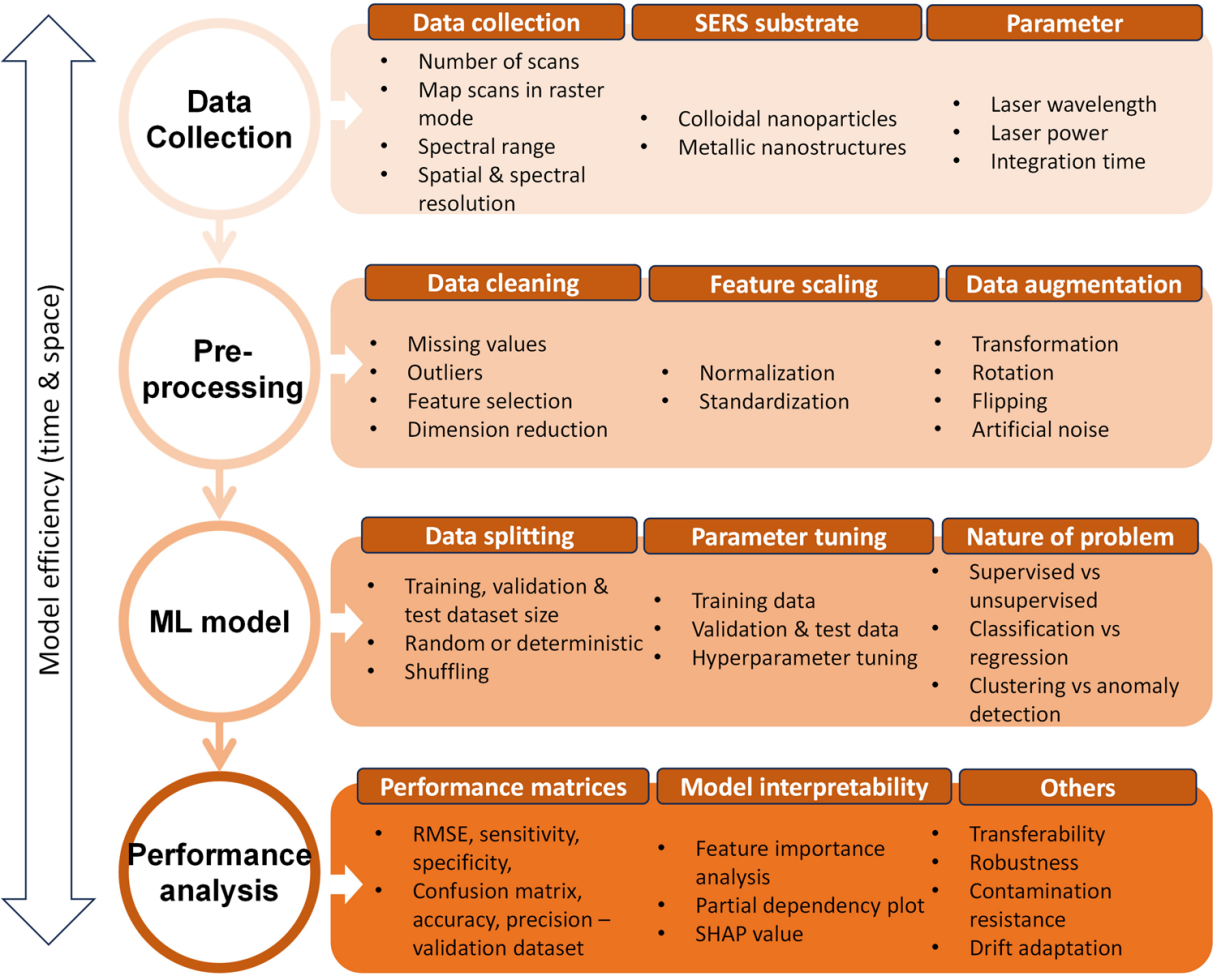
Step-by-step guide for harnessing SERS-ML analysis model from SERS data collection to preprocessing and further ML model development and performance analysis.

Following the collection of SERS data, the process of constructing an ML model encompasses three major steps: data preprocessing, model development, and performance analysis. Figure 1 outlines a step-by-step process to develop a ML model for SERS data. Data preprocessing and cleaning is a fundamental step for SERS-ML models. Raw SERS spectra require cosmic ray removal, graph smoothening, and background subtraction of fluorescence or electromagnetic interference, substrate impurities, or electronic noise.^94^ Preprocessing also includes handling outliers and addressing inconsistencies or errors in the data set. Normalization or standardization is crucial for SERS data to eliminate variability and ensure accurate comparison. Additionally, feature selection or dimensionality reduction using PCA or latent vectors improves model efficiency and interpretability.

After preprocessing the data, the next step in supervised ML model development involves dividing the data set into training, validation, and test sets. Hundreds to thousands of SERS spectra can be obtained from replicates and through large-area scans in SERS. These data can be used for training and testing. Following this step, an appropriate ML algorithm or model architecture is selected based on factors such as the nature of the problem (e.g., classification, regression), the characteristics of the data (e.g., structured, unstructured), and the desired outcome (e.g., accuracy, interpretability). Although there is no definitive method for selecting a specific algorithm over others, exploring various approaches is appropriate for selecting the best fit. Using the selected algorithm, the model undergoes training on the training set, iteratively adjusting parameters to minimize a specified loss function, commonly achieved through gradient descent optimization.^95^ Model performance is evaluated on the validation set using metrics such as accuracy, precision, recall, F1 score, or mean squared error to gauge generalization to unseen data. Hyperparameters are adjusted, or alternative models are explored to improve results. Final performance is tested on the test set for an unbiased real-world estimate, with iterative refinements based on validation and test outcomes.

Choosing a machine learning model for large, complex data sets involves more than performance metrics. For real-life applications, researchers should also consider algorithm efficiency, balancing accuracy with the time and space needed for training and deployment. It is also essential to evaluate the complexity of data relationships (whether linear or nonlinear) and the size of the data set to prevent overfitting or underfitting. This can be achieved through bias-variance analysis and by examining learning curves.^96^ Effective hyperparameter tuning, using techniques such as GridSearchCV or RandomSearchCV, ensures optimal performance without excessive computation.^97^ Finally, interpretability is crucial for understanding how models make decisions, particularly when detecting environmental contaminants. Tools for feature importance analysis such as Partial Dependence Plots, and SHAP (SHapely Additive exPlanations) values can help clarify each feature’s contribution, ensuring greater transparency in the decision-making process.^98^

### 3.2 Why ML for SERS Analysis?

SERS generates abundant data, posing substantial challenges in terms of comprehension and analysis. Within the span of a few seconds to minutes, comprehensive SERS data sets can be collected for individual molecules. Additionally, multicapture SERS platforms excel at identifying the unique vibrational fingerprints associated with target molecules, offering detailed spectral information.^99^ However, when employed for real environmental matrices, SERS encounters significant challenges. The presence of contaminants in environmental samples is characterized by extremely low concentrations, thus posing challenges for detection through analytical methods alone. Another major issue arises from the diverse array of coexisting compounds in real samples, potentially masking the signals of target molecules and making it difficult to discern subtle changes through visual inspection. This necessitates a robust analytical tool capable of not only handling voluminous data but also conducting multiplex analysis with high sensitivity and identifying complex relationships. Another significant advancement in SERS-ML analysis lies in considering multiple peaks for multiplex data sets. Whether labeled or label-free, SERS requires the comparison of spectral intensities against target concentration for subsequent analysis and quantification. The conventional method for analyzing SERS data often focuses solely on a univariate assessment of a single vibrational mode peak intensity and examining its trend across varying target concentrations. In cases with multiple targets or reporter molecules, peaks corresponding to each molecule are selected. Although this approach is effective in simple conditions with minimal background interference, it may be insufficient when multiple targets are present, as variations in peak intensity may no longer be solely influenced by changes in concentration. Choosing multiple peaks for the same molecule allows for more robust quantification compared to relying on a single peak.^93,100^ The multiple peaks assignment requires multivariate analysis and paves the way for employing ML for more comprehensive analysis.

Conventional methods often focus solely on a univariate assessment of a single vibrational mode, examining intensity changes that correlate with alterations in concentration. While this approach demonstrates effectiveness in simple scenarios involving one or two analytes, its limitations become evident in complex systems. ML enables analysis using multiple or entire spectra, thereby providing a more accurate and robust analysis.^101^ Furthermore, the integration of ML into a data analysis flow may aid in uncovering hidden trends. As such, ML is a promising method for extracting previously undisclosed patterns from acquired data, a task that is impossible manually. Additionally, ML can extract peak features crucial for developing models that effectively discriminate between contaminants, thereby expanding the analytical capabilities of SERS in environmental monitoring and beyond. Monitoring pathogens or contaminants in various environmental matrices, such as drinking water and wastewater, using SERS creates opportunities for prompt prevention and enables real-time decision-making for appropriate treatment. When detecting target analytes within environmental matrices, SERS intensities from the background or other molecules can interfere with target spectra, making it difficult to detect the target. The use of ML has shown potential to tackle all these problems, making it versatile and applicable to SERS under complex environmental conditions. Herein, we focus on the detection of biological contamination such as bacteria, viruses, emerging (in)organic contaminants, and microplastics using SERS in combination with ML methods.

## 4 SERS-ML in the Detection of Biological Contaminants

This section delves into the application of SERS-ML for the detection and characterization of biological contaminants in water and wastewater, encompassing (1) bacteria, (2) viruses, and (3) their nucleic acids (DNA/RNA). Through the synergy of SERS’s unique spectral fingerprints and the analytical power of ML algorithms, researchers have made significant progress in augmenting the speed, accuracy, and sensitivity of microbial detection, thereby advancing our understanding of environmental health and safety.

### 4.1 Bacteria

**Figure 2:**
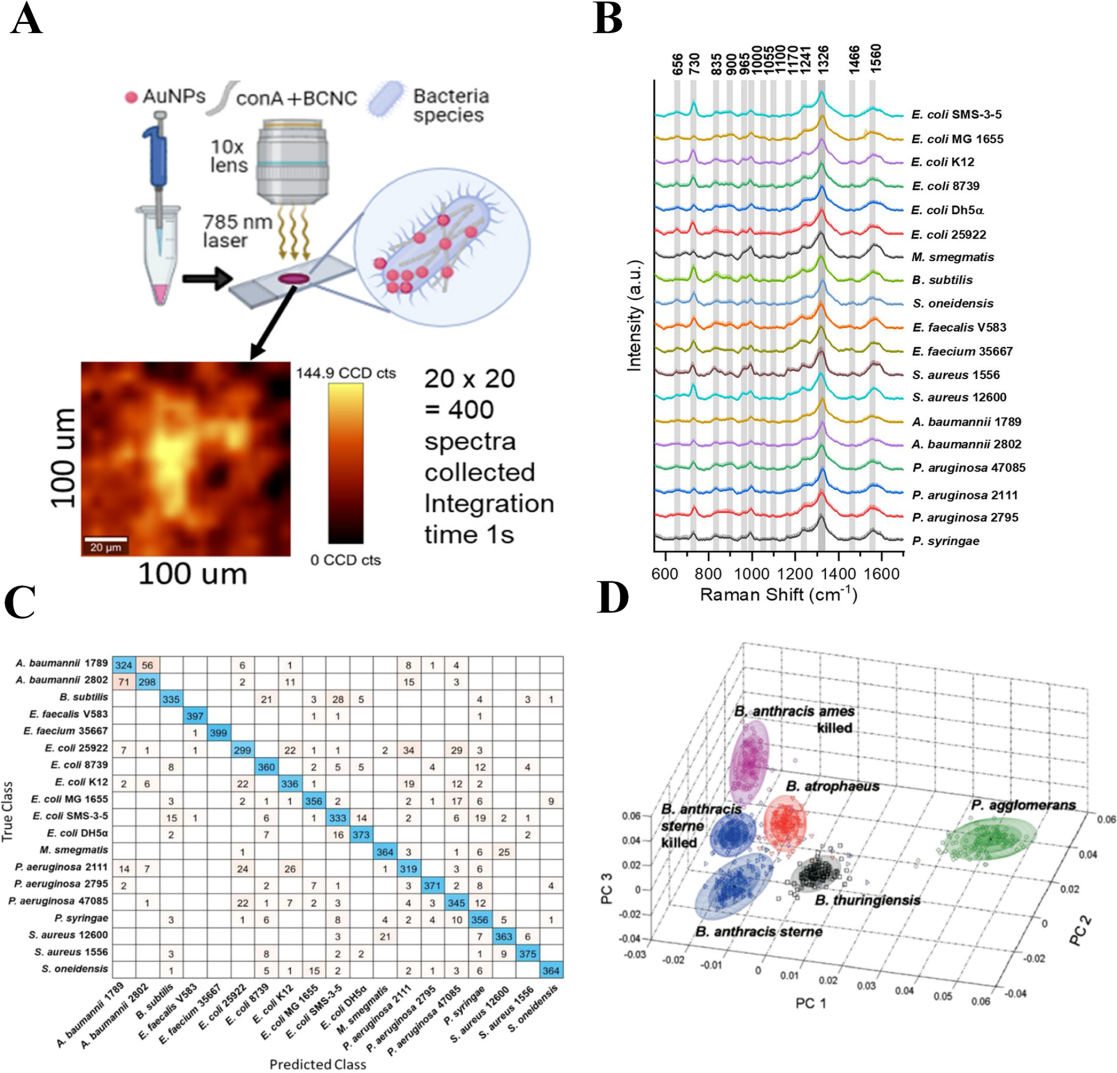
Machine Learning tools for the classification of bacteria using SERS. **A**. Schematic of the Experimental Setup for Label-Free SERS Detection of Bacterial Strains Using Gold Nanoparticles. **B**. Average SERS spectra for 19 bacterial strains, normalized using the peak at 1326 cm^–1^. **C**. Confusion matrix for 19 bacterial strains. Diagonal entries indicate class accuracies (out of 400 spectra), while off-diagonal entries represent misclassifications for each strain. Adapted with permission from ref (74). Copyright 2022 American Chemical Society. **D**. Three-dimensional PCA plot using first three PCs for classification of five Gram-positive *Bacillus* spores (*B*. *atrophaeus*, *B*. *anthracis sterne*, *B*. *thuringiensis*, *B*. *anthracis sterne killed*, *and B*. *anthracis ames killed*) and *Pantoea agglomerans* based on their SERS spectra. Adapted with permission from ref (57). Copyright 2008 Optica Publishing Group.

SERS has emerged as a rapid and reliable approach for bacteria detection that leverages its capability to provide unique spectral fingerprints attributed to the cellular components of bacteria, including nucleic acids, cellular walls, and membranes (Table S1). However, the similarity of the collected SERS spectra makes it difficult for effective differentiation. For example, Rahman et al. reported SERS spectra for 19 different bacteria strains by mixing them with gold nanoparticles. (Figure 2A,B).^74^ The prominent peaks associated with bacteria typically arise from the ring vibrations of adenine and guanine, occurring at 600–735 and 1325 cm^–1^.^74^ The variations in peak ratios, intensities, and shifts in wavenumbers are too subtle to discern manually. Hence, the complexities of SERS spectra necessitate the application of machine learning for thorough and accurate analysis.

By harnessing the power of ML models, we can unravel subtle nuances in SERS spectra, enabling us to not only distinguish between different bacteria but also quantify their presence more accurately. Over the years, SERS-ML has become popular in the identification of bacteria at the species and strain level. Classification ML models such as PCA, SVM, and HCA have been extensively used for the diagnosis and differentiation of biological samples. Several studies have used PCA for classifying closely related strains such as *cereus group Bacillus strains*,^56^*anthracis sterne* live or killed,^57^ serotypes of *salmonella*,^102^ and many more. In a recent study, Arslan et al. discriminated *Cryptosporidium parvum oocysts*, *E*. *coli*, and *Staphylococcus aureus* using PCA and HCA.^59^ By using medium Gaussian SVM model with 10-fold cross-validation, Rahman et al. were able to predict bacterial strains with 87.7% accuracy as shown in the confusion matrix (Figure 2C).^74^ Guicheteau et al. classified *Bacillus spores* using three PCs, which accounted for greater than 90% of the variance in the SERS spectra as shown in Figure 2D.^57^ Gram-negative bacterium *Pantoea agglomerans* showed good separation from Gram-positive *Bacillus spores*. However, among Gram-positive *Bacillus spores*, there is an overlap between the three strains of *Anthracis* as they share the same exosporium layer.^57^ It is interesting to note from above-mentioned studies that PCA was effective in the classification of Gram-positive and Gram-negative bacteria. The reason is the large variance in their SERS spectra because of different cell wall structures. Gram-negative bacteria such as *E*. *coli* possess a lipid-rich outer cell well and a thin peptidoglycan layer. However, Gram-positive bacteria such as *S*. *aureus* have thicker peptidoglycan cell walls than Gram-positive bacteria.^57^ Parasites such as *C*. *parvum* oocysts have much different cell structures than bacteria^59^ and contain high carbohydrate components within the wall structure. Here, PCA and HCA are effective in classification since the linear combination of spectra features differentiates the targets. However, while the differentiation of two bacteria from the same categories has very similar spectral features, PCA does not do a good job of classifying complex and nonlinear data. Most of the time, PCA is used for dimension reduction, and the PCs are then used as inputs for advanced machine learning techniques such as SVM.

Wang et al. used multiplexing to detect waterborne pathogens using labeled SERS with SVM.^103^ Three bioconjugated gold nanoparticles were used to specifically bind *E*. *coli O157:H7* cells at different epitopes. To differentiate between positive signals and negative signals, SVM was employed using the input of the first 58 PCs. A linear kernel was used in the SVM and showed clear differentiation between positive and negative spectra with a limit of detection of 10 CFU/mL. Another study classified three antibiotic resistance isolates of *E*. *coli ATCC25922*, *E*. *coli ST131:O75*, and *E*. *coli ST1193:O25* following direct deposition on a gold nanoparticle substrate. Despite having very similar SERS spectra, the SVM-PCA-assisted model showed excellent performance in classifying these strains.^104^

SERS-ML promises to revolutionize the field of bacterial detection, offering a faster, more cost-effective, and more precise alternative to traditional methods, thereby advancing our ability to understand and combat microbial threats in diverse environmental samples.

### 4.2 Virus

Wastewater monitoring for virus detection has been done since the outbreak of poliovirus.^105^ Viruses shed in the fluids of symptomatic or asymptomatic patients enter wastewater systems and may remain infectious for up to 30 days.^106^ To prevent the outbreak of viral disease, it is crucial to detect viruses early and take preventive actions. Moreover, since late 2019, the coronavirus SARS-CoV-2 has spread quickly worldwide thus demanding rapid point-of-care testing (POCT) methods to facilitate early diagnosis outside the laboratory. Conventional methods such as ELISA and reverse transcription PCR (RT-PCR) analysis have been used to diagnose viruses, and typically have high sensitivity (for SARS-CoV-2 RT-PCR can achieve 500–1000 copies/mL of viral RNA).^107^ Alternatively, without compromising high specificity and sensitivity, SERS has been used for detecting viruses at concentrations as low as 80 copies/mL within an hour.^108^ Zhang et al. employed bromide-coated silver nanoparticles and Ca(II) ions as aggregates to enhance hotspot formation, ideal for detecting viruses of 100 nm diameter.^60^ SARS-CoV-2, Human Adenovirus 3, and H1N1 Influenza virus exhibited similar SERS spectra and were more challenging to differentiate in complex media such as serum and saliva, the authors utilized PCA to facilitate effective discrimination. By using PCA, they successfully detected viruses at a concentration of 100 copies per test, within 2 min. While PCA captures maximum variance in the data, it does not consider class labels unlike supervised ML models, and may not be very effective in maximizing separation between different classes.^48^ A similar study led by Garg et al. used a supervised PCA-LDA model to differentiate different enveloped viruses.^109^ Their approach involved utilizing PCA for feature extraction and dimension reduction, followed by LDA to identify a subspace that optimally separates different classes within the data. They were able to differentiate SARS-CoV-2, Zika, and Influenza A viruses within an environmental dust background with 86% accuracy. While these models demonstrated satisfactory performance, their limitations include their capability to detect viruses at very low concentrations (<100 copies) and the absence of cross-validation.

To detect extremely low concentrations of viruses, Yang et al. developed a hierarchical array of gold nanoneedles (GNAs) functionalized with angiotensin-converting enzyme 2 (ACE2).^108^ ACE2, known for its high specificity to SARS-CoV-2, effectively captured and enriched the viruses near the GNA hotspot region. By incorporating PC-discriminant analysis of SERS signals, they achieved the detection of viral loads as low as 80 copies/mL in under 5 min. While the ACE2-based method demonstrates high specificity for SARS-CoV-2 it exhibits limitations in detecting viral variants. A study led by Moitra et al. developed a set of DNA probes, specifically an antisense oligonucleotide (ASO) capable of interacting with genetic sequences of SARS-CoV-2 regardless of its mutations. Thiolated ASOs targeting the N gene of SARS-CoV-2 were attached to gold nanoparticles, allowing for the examination of viral RNA attachment to ASOs and producing strong SERS signals. With the combination of SERS with the PCA-SVM model, they were able to predict positive samples with 100% sensitivity and 90% specificity at concentrations up to 63 copies/mL of RNA. This demonstrates that utilizing the indigenous design of SERS probes alongside ML techniques enables real-time detection of viral variants without the need for sophisticated instrumentation.

An alternative method for virus or bacteriophage detection involves analyzing alterations in SERS signals emitted by bacteria or their metabolites postinfection. In a study led by Wang et al., they determined that dimethyl disulfide (DMDS), a volatile sulfide metabolite, accumulated in the headspace of a sealed Petri dish.^73^ Accumulation of DMDS was monitored using silver nanoparticle-based SERS tape affixed to the dish cover, and subsequent SERS spectra were obtained. Notably, during virus infection (Phi6) of *P*. *syringae*, changes in the SERS intensity of the DMDS peak were observed. To discern the statistical distinction between infected and noninfected bacteria, a PCA-SVM classification model was employed, utilizing a quadratic kernel and the first nine principal components (accounting for 95% variance). The classification confusion matrix revealed an overall accuracy of 93%, with sensitivity and specificity exceeding 92%. These findings suggest that instead of solely examining virus-specific SERS peaks, bacterial peaks or metabolites can serve as indicators of viral presence. Despite subtle changes, ML models offer improved classification capabilities.

### 4.3 Nucleic Acids

Extensive research has been conducted in the identification of nucleic acids to characterize microbial communities in drinking water, wastewater, and soil both as a means to understand the impact of environmental parameters on microbial communities^110^ as well as to detect pathogenic DNA/RNA^111^ and antimicrobial resistance genes.^112,113^ Nonenzymatic methods, including DNA microarrays, nanopores, and mass spectrometry, have emerged as rapid and effective tools, enhancing the sensitivity and specificity of DNA sequence detection. However, the noninvasive detection of SERS surpasses these methods in terms of sensitivity, speed, and simplicity.

**Figure 3:**
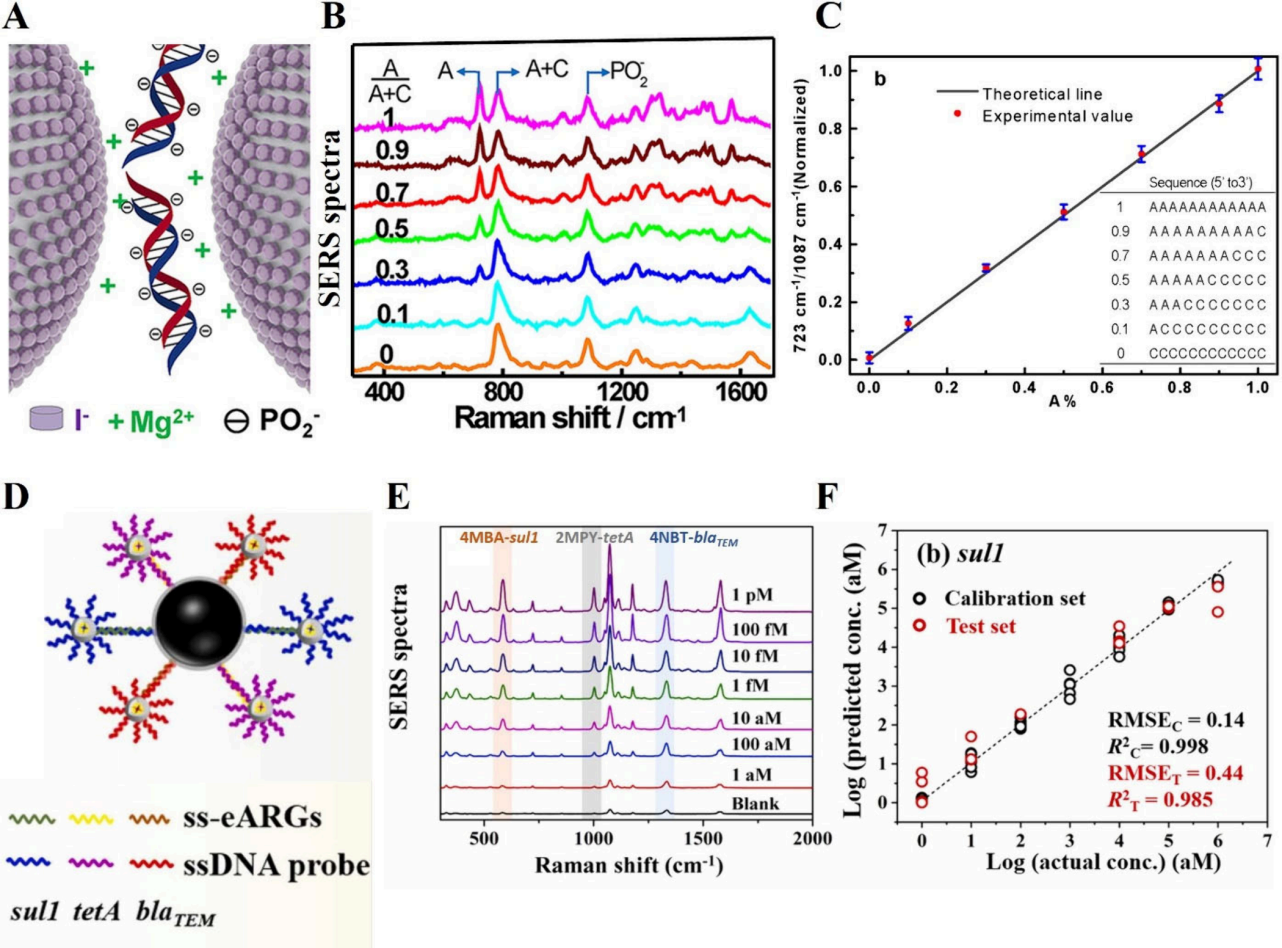
Detection of DNA using SERS **A**. Illustration of iodide-modified silver nanoparticles along with MgSO_4_ to enhance DNA binding. **B**. SERS spectra of a series of oligonucleotides containing varying proportions of adenine (A) and cytosine (C), where A% = A/(A + C). All spectra are normalized to the peak intensity at 1087 cm^–1^, corresponding to PO_2_^–^. **C**. Plot of relative peak intensity ratio of 723 cm^–1^ to 1087 cm^–1^ as a function of A% in the oligonucleotides. Adapted with permission from ref (117). Copyright 2015 American Chemical Society. **D**. Schematic illustration of the core–satellite SERS sensor for detection of multiple eARGs (extracellular antibiotic resistance genes). **E**. SERS spectra of multiple eARGs in the range from 1 aM to 1 pM. **F**. Comparison of *sul1* concentration for actual vs predicted. Black dots represent the calibration set (80% of the data set) and red dots represent the test set, comprising the remaining 20%. Adapted with permission from ref (69). Copyright 2022 Elsevier.

Nucleic acids exhibit distinctive fingerprint information that reflects their breathing and ring skeleton vibration modes and they are well-suited for label-free SERS detection.^114^ The Bell group^115,116^ has successfully detected DNA and RNA using silver nanoparticles and MgSO_4_. The presence of MgSO_4_ as the aggregation agent induces the formation of nanoparticle/nucleotide aggregates, resulting in an increased number of hotspots. Consequently, such natural trapping of nucleic acids significantly enhances the signal strength. Xu et al. used iodide-modified silver nanoparticles and MgSO_4_ to neutralize the surface charge and enhance DNA binding as shown in Figure 3A.^117^ The authors demonstrated the effectiveness of using the phosphate backbone (PO_2_^–^) as an internal standard for single-base DNA analysis through SERS. By synthesizing oligonucleotides with varying adenine (A) and cytosine (C) ratios, they normalized the SERS spectra based on the intensity of PO_2_^–^ (Figure 3B). This revealed a clear, linear trend in the characteristic adenine band intensity against increasing concentration. Moreover, plotting the relative SERS intensity of the adenine peak (723 cm^–1^) to the PO_2_^–^ peak (1087 cm^–1^) against the A/(A + C) ratio resulted in a remarkably linear relationship (Figure 3C). This method demonstrates high precision and sensitivity for single-base discrimination. However, label-free detection encounters a significant challenge stemming from the similarity of the collected SERS spectra that can be attributed to the common phosphate/sugar backbone shared among nucleic acids. Raman peaks derived from phosphate, notably those at 815/860, 1087, and 1230 cm^–1^ represent the symmetric bend, symmetric stretch, and asymmetric stretch modes of PO_2_^–^ and contribute to the overall spectral resemblance of numerous nucleic acids.^117^ Typically the only readily detectable difference lies in the relative intensities of various nucleobases. Consequently, distinguishing between nucleotides is challenging, especially at lower concentrations. One effective approach to overcome this challenge is to employ multivariate analysis. PCA in combination with DA has been used to classify different nucleic acids.^118^ Furthermore, achieving single-base sensitivity or distinguishing mismatches poses a challenge with SERS alone. Kang et al.^100^ addressed this issue by employing tree-based multiclass support vector machine (Tr-SVM) classifiers to differentiate SERS spectra of gene sequences with 2–10 base mismatches. A tree-based decision rule was utilized to group correlated classes, offering multiple classifiers based on one of two decision levels. SVM was applied to maximize the margin between different classes using an optimal hyperplane. Through 10-fold cross-validation, this adaptable discriminatory tool accurately identified antibiotic resistance genes with a prediction accuracy of 90%.

Alternative to label-free detection, an indirect method that consists of the hybridization of nucleic acids can also be used to detect DNA/RNA. In this approach, SERS probes containing a complementary strand and a Raman reporter hybridize with a target, increasing the Raman reporter’s intensity.^119^ Research employing such a hybridization technique with a SERS probe can identify the presence of nucleic acids at concentrations in the picomolar^120^ to the femtomolar range.^121^ However, concentrating solely on a change in the intensity of a single peak in the presence of a target gene may be affected by background interference, particularly in complex matrices. Lu et al. identified extracellular antibiotic resistance genes (eARGs) including *sul*1, *tet*A, and *bla*_TEM_ through a hybridization approach using the Raman reporters 4-mercaptobenzoic acid (4-MBA), 2-mercaptopyrimidine (4-MPY), and 4-nitro blue tetrazolium chloride (4-NBT) in environmental samples (Figure 3D).^69^Figure 3E illustrates SERS spectra for eARGs across concentrations ranging from 1 aM to 1 pM. The sensitivity of this method was compromised by the complexity and signal attenuation induced within environmental samples such as wastewater treatment plant effluent, aquaculture water from cattle farms, and groundwater. To reduce noise interference, unsupervised ML for multivariate analysis was used by selecting multiple characteristic peaks of Raman reporters from the total SERS spectra hence, increasing robustness compared to univariable analysis. Among SVM, RF, PLS, and multilayer perceptron (MLP), RF was most suitable with the lowest root-mean-square error (RMSE) and highest regression coefficient (*R*^2^) shown in Figure 3F for *sul1*. The concentration of eARGs in environmental samples, determined through RF, was comparable to that of ddPCR with no statistically significant difference (*p-value* > 0.05).

## 5 SERS-ML for the Detection of Organic Pollutants

This section explores the application of SERS-ML for detecting and characterizing organic pollutants, with an exclusive focus on PAHs and OPPs. Both classes of compounds are toxic, carcinogenic, and persistent in various ecosystems, making their detection critical for environmental monitoring. Given the complexity of environmental matrices, detecting PAHs and OPPs at the trace level presents significant analytical challenges. By focusing on these pollutants, we aim to highlight the effectiveness of SERS coupled with ML in overcoming these challenges and provide precise classification and concentration of pollutants.

### 5.1 PAHs

PAHs are a hazardous class of chemicals whose structure consists of multiple fused benzene rings.^122^ PAHs have sparked significant environmental concern due to their carcinogenic and mutagenic characteristics, and their propensity to readily pollute essential natural resources such as drinking and river water.^123,124^ These compounds arise through the incomplete combustion of coals and fuels,^125,126^ resulting in their existence not as isolated chemicals, but as complex mixtures. This complexity poses a challenge for the on-site identification of PAHs. The detection of PAHs using SERS poses several challenges. One of them is that the hydrophobic nature of PAHs inhibits their adsorption to citrate-stabilized Au or Ag colloids due to their incompatibility with the surface chemistry.^127^ Attempts have been made to overcome this limitation by modifying the substrate surface with colloidal hydrophobic films.^128,129^ Surface modifications such as thiol-modified and oleate-modified Fe_3_O_4_@Ag microspheres resulted in a 10^–8^ mol/L limit of detection (LOD).^127^ Another challenge lies in accurately quantifying trace concentrations of PAHs, as spectra often contain multiple analytes with overlapping peaks, varying signal-to-noise ratios, and significant background interference. Incorporating ML methods with SERS has shown the possibility of detecting PAHs as low as 5 nmol/L in PAH mixtures because of its ability to handle the nonlinear relationship between concentration and spectrum intensity.^130^ Atta et al. employed two one-dimensional CNNs for the multiclass classification and regression analysis of SERS spectra.^86^ The CNN models, optimized through training on a calculated data set, demonstrate high precision (97%), F1 score (94%), and accuracy (90%) in classifying pollutants. The CNN regression model effectively predicted pollutant concentrations, achieving a combined *RMSE*_*spectrum*_ of 5.92 × 10^–2^ and *RMSE*_*conc*_ of 1.07 × 10^–1^ (μM). In complex scenarios, where spectral data from the same class varies over time and overlapping peaks (as seen here), more sophisticated algorithms such as CNNs are more effective than basic algorithms. The effectiveness arises from their ability to not only extract features, but also capture various patterns and possibilities, resulting in improved performance in complex analyses.^25^ In addressing the challenge of overlapping spectra arising from structurally similar benzene structures in PAHs, a recent investigation led by Bajomo et al. introduced an innovative unsupervised machine learning method named Characteristic Peak Extraction (CaPE) algorithm for dimension reduction to extract distinctive SERS peaks of PAH mixtures.^131^ By analyzing SERS spectra from complex mixtures, particularly for varying concentration ratios, CaPE efficiently identified and extracted spectra of individual components, subsequently matching them against an SERS spectral library for identification. By integrating chemical sensing with the CaPE algorithm, they were able to effectively address challenges such as incomplete libraries and frequency shifts in SERS peaks. The SERS-ML tandem methodology exhibits significant potential for rapid, on-the-field identification and detection of chemicals based on molecular structures, outperforming conventional demixing algorithms.

### 5.2 OPPs

**Figure 4:**
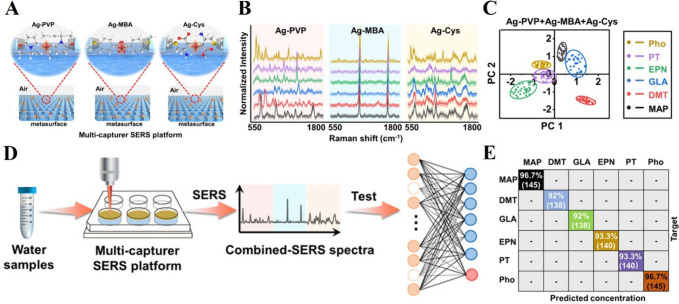
Detection of organophosphorus pesticides (OPPs) in water using a deep-learning-based multicapture SERS platform. **A**. Schematic diagram of PVP, MBA, and Cys stabilized Ag nanocubes at the air/water interface and **B**. their corresponding SERS spectra for each OPPs (MAP, DMT, GLA, EPN, PT, and Pho). **C**. PCA plot showing cluster separation with multicapturer Ag-PVP + Ag-MBA + Ag-Cys. **D**. A schematic illustrating the detection of OPPs in environmental water samples, combining SERS spectra collection with a developed deep learning model. **E**. Confusion matrix showing the regression results for OPP concentration predictions in environmental samples. Adapted from ref (93). Copyright 2022 American Chemical Society.

Another emerging contaminant class in environmental systems, primarily sourced from agricultural runoff, are OPPs such as methamidophos (MAP), dimethoate (DMT), parathion, diazinon, and others. The potential toxicity of OPPs poses serious health risks, including acute and chronic neuropathy, reproductive toxicity, and endocrinopathy, highlighting the need for vigilant environmental monitoring. Several studies have been done on the detection of OPPs using SERS -ML either using a label-free strategy^132−134^ or by leveraging the interaction between OPPs and reporter molecules (e.g., 4-MBA, l-cysteine) attached to nanoparticles, leading to a change in SERS spectra. Li et al. conducted a study using plasmonic nanocube metasurfaces (NCMs) to detect various OPPs, including MAP, DMT, glufosinate ammonium (GLA), ethyl para-nitro-phenyl (EPN), parathion (PT), and phosmet (Pho), achieving multiplex determination (Figure 4).^93^ Potential affinity agents for OPPs consist of poly(vinylpyrrolidone), 4-MBA, and l-cysteine assembled on Ag nanocubes self-assembled at liquid/liquid interface (Figure 4A). The combined SERS spectra of OPPs were reconstructed (Figure 4B), enhancing the spectral variations for each OPP. SERS spectral variances before and after the capture of OPPs on the modified NCMs were complex and could not be distinguished manually. Hence, PCA was used to extract the spectral variances (Figure 4C) and as an input for a ResNet-deep learning model. The model demonstrated a classification accuracy exceeding 96% and a regression accuracy surpassing 92%. Furthermore, the model successfully identified all six OPPs spiked in environmental water samples (farm, river, and fishpond water), highlighting the capability to exclude interference from other matrices in real environmental samples (Figure 4D). The regression confusion matrix for concentration predictions demonstrated an accuracy exceeding 92% (Figure 4E).

## 6 SERS-ML in the Detection of Inorganic Pollutants

In this section, we explore the intersection of water quality monitoring and advanced analytical techniques, focusing on the detection of inorganic anions and cations.

### 6.1 Anions

Regular monitoring of water systems is essential due to the occurrence of problematic inorganic nitrogen and sulfur species, such as nitrates, nitrites, and sulfates. When these compounds are present in elevated concentrations, they can pose substantial risks to aquatic life and other organisms. For instance, nitrates and nitrites can induce eutrophication and disturb aquatic ecosystems, whereas sulfates may aid in the creation of sulfuric acid, leading to pH declines in water bodies and subsequent disruptions.^135^ Anthropogenic sources of nitrates in water and wastewater primarily reflect agricultural discharge coming from pesticides resulting in eutrophication^136^ and elevated concentrations of nitrite can cause blue baby syndrome in infants.^137^ SERS being a less destructive and noninvasive technique can help with the *in situ* detection of these anions. The inherent negative charge of anions elicits charge–charge repulsion, however, limiting the application of SERS. To address this, cation-coated nanoparticles and SERS substrates have proven effective in mitigating the repulsive forces and enhancing SERS capabilities. Moiser-Boss et al. employed a cationic silver substrate to detect nitrates and sulfates via solid-phase extraction.^138^ They successfully detected nitrates at concentrations below 100 ppm. Kuster utilized nanostructured gold substrates without surface functionalization for the *in situ* detection of nitrate.^139^ PCA was employed to time-averaged SERS spectra over the range of 1000 cm^–1^ to 1350 cm^–1^ to distinguish varying concentrations of nitrates in water through SERS. This method proved effective in achieving improved differentiation, considering the spectral noise measurements.^140^ Gajaraj and colleagues identified the presence of nitrate in water and wastewater at a low concentration of 1 mg/L.^141^ However, when analyzing actual wastewater samples, the absorption of nitrates in the “hotspot” region was impeded due to the interference of other ions, namely chloride and phosphate. This interference resulted from the competitive interaction among these ions due to their identical charge and ionic radii.^138^ To eliminate chloride adsorption, one method involves applying a coating to the SERS substrate with enhanced selectivity toward nitrates and sulfates.^142^

Until now, limited research has combined ML with SERS for inorganic pollutant detection. While SERS has successfully achieved low limits of detection with single peak observation for anions, addressing complex samples with diverse anions requires more than manual differentiation based on a single peak. Integrating ML holds promise for facilitating multiplex detection of ionic anions in water and wastewater within minutes, streamlining procedures, and significantly improving time efficiency.

### 6.2 Heavy Metal Cations

**Figure 5:**
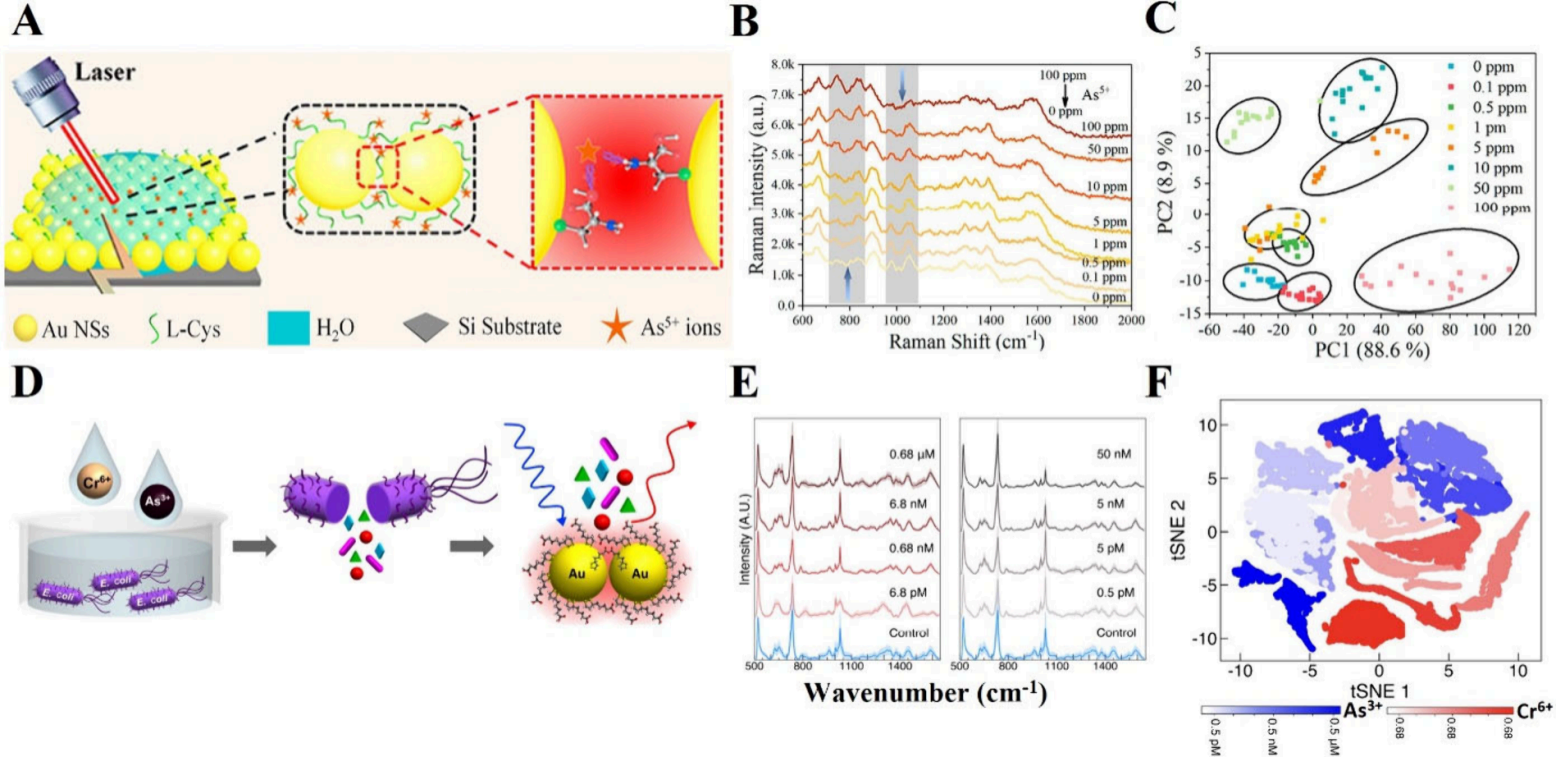
**A**. Schematic illustration of an AuNP/l-cysteine SERS substrate used for detecting As(V). **B**. SERS spectra of As(V) solutions at varying concentrations on the AuNP/l-cysteine substrate. **C**. PCA scatter plot showing the first two principal components for different concentrations of As(V). Adapted from ref (155). Copyright from 2023 American Chemical Society. **D**. Schematic diagram illustrating heavy metal detection using SERS spectra of key metabolites of *E*. *coli*. **E**. Averaged SERS spectra obtained from *E*. *coli* cultured in media with K_2_Cr_2_O_7_ and NaAsO_2_, varying in concentration. **F**. SVM model used to classify between Cr(VI) and As(III) for concentration range 0.68 pM to 0.68 μM and 0.5 pM to 0.5 μM respectively and tSNE clustering analysis for different concentrations of Cr(VI) and As(III) in red and blue, respectively. Adapted from ref (157). Copyright from 2023 Proceedings of the National Academy of Science.

Expanded industrial activities have led to widespread heavy metal pollution in both water and soil.^143^ Accumulation of heavy metals in water and crops poses a significant threat since these materials are potentially toxic to living organisms.^144^ The need to detect metal ions in water has driven advancements in analytical techniques. Direct detection of monatomic metal ions using SERS is challenging due to their small Raman scattering cross-section and the trace concentrations present in water.^145^ To overcome this limitation, metal ions can be detected through chemical binding to organic receptor ligands that induce perturbations in SERS intensities upon the formation of a coordination complex.^146−148^ Chelating agents such as Schiff base,^149^ cyanide,^148^ terpyridine derivatives^147^ have been used for the detection of Co(II),^147^ Cd,^150^ Hg(II),^151^ As(III),^152^ and Cu(II)^146^ with LODs (μg/L) lower than the WHO defined limits. Docherty et al.^149^ identified toxic metal ions including Ni(II), Cu(II), Mn(II), and Co(II) through SERS using [O, N, N, O] tetradentate bis-Schiff (salen) base ligand. The interaction between the salen ligand and various metal ions led to modifications in the intensity and frequency of several bands that was influenced by the size, mass, and coordination bond strength of the metal ions.^153^ Notably, alterations in the C=N stretch peak around 1600 cm^–1^ of the salen ligand were observed introducing different metal ions. It is essential to highlight that although subtle changes are discernible, the identification of peak shifts and intensity changes in complex sample matrices is challenging. The application of PCA proved effective in distinguishing between different metal ions, where each metal ion formed distinct clusters, facilitating clear differentiation. However, it is worth noting that predicting low concentrations of metal ions in real samples posed difficulties due to interfering signals from organic matter.^154^ To overcome the interference from the background signal, Fang et al.^155^ integrated CNN with SERS for predicting the As(V) concentration in water using the chelating agent l-cysteine as shown in Figure 5A,B. PCA was applied to highlight the spectral differences, revealing clear clustering of the spectral data, with the first two PCs accounting for 99% of the variance (Figure 5C). However, significant overlap in clusters was observed at lower concentrations of As(V). When PCA–PLS was used for prediction, the low coefficient of determination (R^2^ = 0.884) and high RMSE of 18.34 indicated poor prediction accuracy, likely due to the complex variables in the SERS spectra. In contrast, combining CNN with SERS provided rapid and accurate predictions of As(V) ions down to 1 ppm, achieving an R^2^ of 0.991 and a much lower RMSE of 3.36. The superior performance of CNNs compared to PCA–PLS may be attributed to the ability of CNNs to handle complex patterns and nonlinear relationships within high-dimensional SERS data.^84^ It is noteworthy that traditional methods like ICP-MS for detecting metal ions can achieve detection levels in the ng/L range,^156^ making them superior to SERS in terms of sensitivity, given that SERS typically has a limit of detection around μg/L. Nevertheless, the SERS method provides a much cheaper and simpler alternative capable of detecting the metal ions at μg/L levels and with ML rapid analysis within a few seconds.

Another compelling study to detect the presence of heavy metals in drinking and wastewater led by Wei et al.,^157^ used bacterial metabolic response transduced by heavy metals into chemical (metabolite) signals using SERS with ML algorithms (Figure 5D). Changes in the SERS spectra of metabolites, specifically the nucleotides ATP, uracil, and adenine, were observed in *E*. *coli* cultures upon exposure to Cr(VI) and As(III) (Figure 5E). The integration of SERS with the SVM model demonstrated a detection limit of 6.8 pM for Cr(VI) and 0.5 pM for As(III), achieving sensitivities and specificities exceeding 97% (Figure 5F). Notably, this approach achieved a LOD 6 orders of magnitude lower than traditional growth inhibition methods relying on optical density. Moreover, the CNN model successfully detected the concentration of heavy metal ions in tap water and wastewater samples, exhibiting the same LOD as the SVM model. This demonstrates that the reliable performance of the SERS model, aided by machine learning, remained stable irrespective of the impurities present in the samples.

In essence, ensuring the quality of drinking water and discharged water necessitates portable, rapid, and highly sensitive detection techniques, a need well-addressed by the combination of SERS with ML.

## 7 SERS-ML in the Detection of Microplastics

Microplastics, tiny plastic particles less than 5 mm in size, typically originating from the breakdown of larger plastic containing items have emerged as a new type of pollutant, and have been detected in air, tap water, river, and seawater.^37,158−160^ The ubiquitous nature of microplastics has raised concern due to their pervasive presence and their potential ecological and human health risks. As these tiny particles infiltrate aquatic ecosystems, soils, and air, detection techniques must adapt to the complexity of plastic sources, their varying sizes, and their concentrations. Several SERS-based direct and indirect-based microplastics detection methods have been published that were able to detect microplastics in concentration ranges from 0.97 ng/mL to 6.5 μg/mL.^161−163^ The similarity in SERS profiles among different microplastics has led to the use of ML methods with the potential to significantly expedite experimental analysis and computation. Luo et al.^164^ introduced an innovative approach combining CNN with SERS for the rapid identification and classification of microplastic mixtures. Six types of microplastics were dispersed in water, creating 6300 sets of SERS spectra for training and testing. The confusion matrix results of each MP of the trained CNN model show that polycarbonate, polystyrene, and polyethylene terephthalate were all identified correctly. The CNN model achieved a remarkable identification accuracy of 99.54% without extensive spectral preprocessing, demonstrating its robustness in handling unprocessed SERS spectra for rapid and accurate identification of complex MP mixtures.

**Figure 6:**
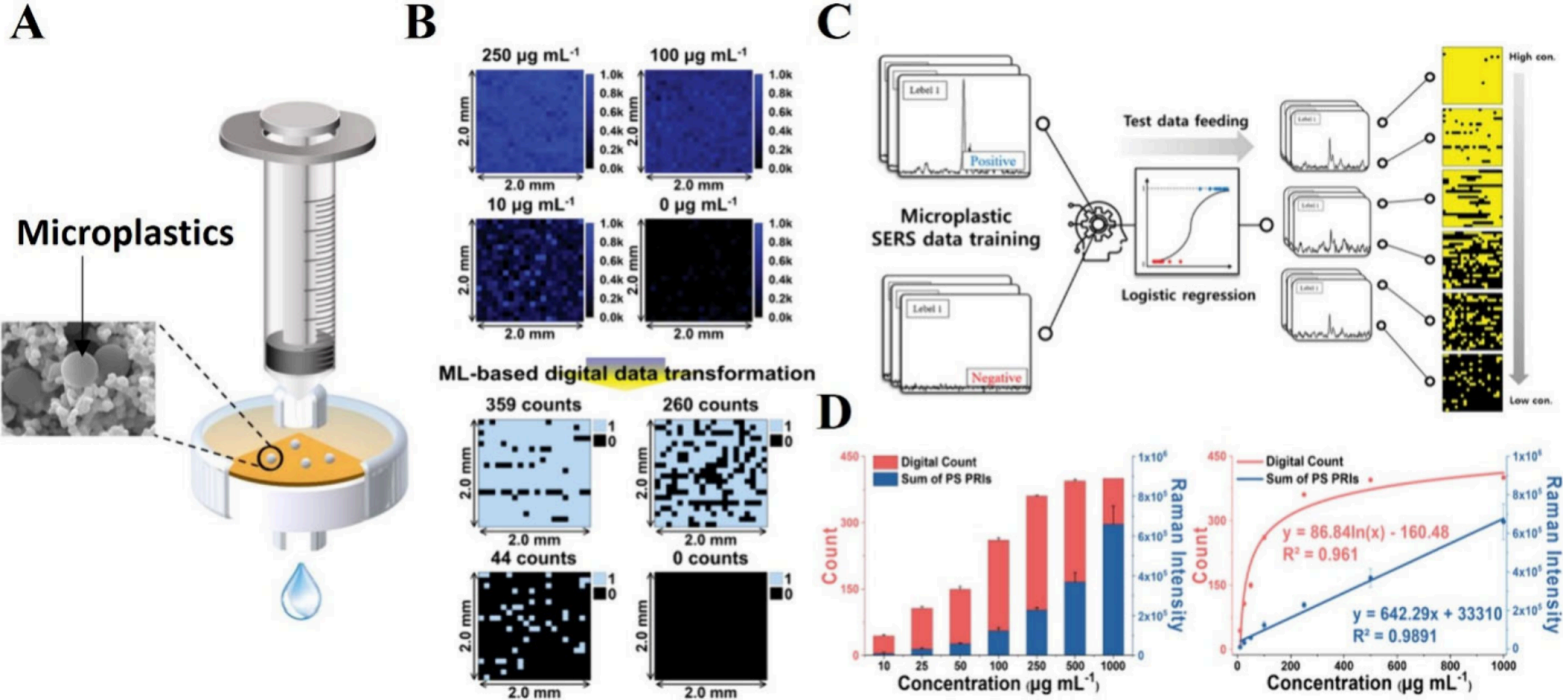
Detection of microplastics using SERS-based logistic regression model. **A**. Schematic illustration of a 3D-PGNP system integrated with a syringe filter for microplastic filtration. **B**. SERS maps for PS at concentrations ranging from 0 to 250 μg/mL, along with the corresponding digital SERS maps after applying ML model. Black pixels represent the absence (0) of PS, while blue pixels indicate its presence (1). **C**. Schematic illustration of microplastic classification using the logistic regression method, including data training, data input, and quantification. **D**. Quantitative digital counts and the sum of positive Raman intensities (PRI) alongside the corresponding calibration curve. Adapted from ref (80). Copyright from 2023 Wiley Online Library.

Recently, Kim et al. developed 3D-plasmonic gold nanopocket (3D-PGNP) nanostructures integrated with a syringe filter to filter and detect polystyrene (PS) and polyethylene (PE) microplastics as shown in Figure 6A.^80^ Using SERS spectra of microplastics along with background noise, a logistic regression model was trained to convert map data into a digital format by classifying pixels as MP-positive or MP-negative (Figure 6B,C). By using digital counts, they were able to detect PS at a concentration of 2.5 μg/mL. When quantifying positive pixels in the digital data, a logarithmic trend is observed between the digital count and the concentration of PS (Figure 6D). Additionally, they applied the developed model to complex environmental samples by spiking microplastics into tap water, river water, and seawater, successfully detecting low concentrations of PS with a high recovery rate, even in the presence of interferences.

## 8 Future Scope: Translating SERS-ML Potential into Real-World Solutions

The integration of SERS with ML holds exceptional promise to revolutionize various fields by offering rapid, sensitive, and robust analytical methods. In environmental monitoring, SERS-ML platforms integrated with sensor networks have the potential to transform pollution monitoring with source tracing, thus enabling targeted mitigation and remediation strategies. Similarly, in biothreat detection, healthcare biosensing, and food safety, SERS-ML technologies offer significant potential. Despite such potential, the widespread adoption of SERS-based sensors as a standard recognition tool faces several challenges, necessitating concerted efforts to address these limitations and unlock future opportunities.

One of the primary hurdles is the lack of standardized protocols for SERS-ML applications. SERS spectra can be influenced by various factors such as substrate morphology, experimental conditions, and complex backgrounds, leading to challenges in interpretation and reproducibility. To address this issue, the development of standardized data sets and protocols, along with incorporation of internal standards or the establishment of calibration curves, is crucial. Identifying relevant spectral features and designing effective feature extraction methods can further improve SERS-ML models.

Addressing fundamental challenges in ML is also pivotal for advancing SERS-ML capabilities. Generalizability remains a significant concern, as models trained on lab-generated data sets may stumble when applied to real-world samples due to complex matrix effects and variations in background interference. Techniques such as meta-learning, adversarial robust ML, and domain transfer can enhance model robustness and out-of-distribution generalization.^165^ Furthermore, the interpretability of ML models is essential for understanding the underlying chemical and physical processes captured in SERS spectra. While complex models such as deep neural networks offer high predictive power, their black-box nature poses interpretability challenges. Leveraging techniques such as explainable artificial intelligence, and visualization tools can help both ML practitioners and domain experts extract meaningful insights from complex spectra, further enhancing trustworthiness. Moreover, transitioning from a model-centric to a data-centric AI approach in SERS-ML is key to tackling real-world challenges more effectively. While conventional methods prioritize models, they often stumble in real-world scenarios due to data complexities. A data-centric focus prioritizes data quality, preprocessing tailored to SERS specifics, and robust validation. This ensures models are trained on top-notch data sets, boosting performance and adaptability. Embracing this shift not only deepens our understanding of data but also equips us with more resilient ML algorithms for practical SERS applications.

While resource-intensive, another hurdle is the requirement for rigorous validation against established analytical techniques on large sample sets as a means to validate the reliability and robustness of SERS-ML methods. Exploring synergies between SERS and other analytical techniques such as mass spectroscopy, infrared spectroscopy, and PCR can enrich chemical analysis and increase the reliability of SERS analysis. Integrating multimodal data fusion approaches can enhance the robustness and accuracy of SERS-based chemical sensing platforms. Collaborative interdisciplinary efforts are key to realizing the transformative impact of SERS-ML technologies on pressing societal and environmental issues.

Finally, the transformation of SERS-ML into a portable, field-ready tool necessitates comprehensive technological advancements at the system level. This entails the creation of compact SERS devices that seamlessly integrate with portable Raman spectroscopy tools and streamlined sample collection and handling systems. The development of efficient ML algorithms, along with optimized distributed controller-computing-communication systems tailored for resource-constrained environments, and the exploration of hardware acceleration solutions, are pivotal needs for enabling on-site, real-time detection and decision-making driven by SERS-ML technology.

## 9 Reflecting on the Potential: A Call to Contemplate SERS-ML Integration

While the authors acknowledge the significant potential of SERS-ML and foresee a promising future for integration, we present key considerations regarding the application of SERS-ML in real-time scenarios. Additionally, we offer a grand vision for transforming the field through the collaborative integration of SERS with ML. The first aspect to contemplate is democratized sensing and decision-making. Advancements in robust AI and hyperspectral analytics stand at the forefront of revolutionizing water quality monitoring. These advancements will enable the deployment of multiparameter aerial and autonomous aquatic robotic systems, equipped with SERS technology, across diverse geographical regions. By incorporating SERS-ML integration, these systems will have the capacity to efficiently detect and analyze water contaminants in real time, offering comprehensive insights into water quality. Cost-effective, miniaturized sensors coupled with interpretable ML software can provide decentralized, real-time insights into water quality for local communities and municipalities.

Continuous scientific discoveries are also anticipated. The integration of vast data sets from distributed monitoring, along with online deep learning and explainable models, may unveil new interdependencies and biogeochemical mechanisms. This continuous enhancement of process and fate knowledge regarding emerging pollutants is crucial for sustaining usable resources and has the potential to establish a feedback loop for diagnostics and future solutions. Moreover, evidence-based interventions and adaptations are envisioned. By combining spatiotemporal high-resolution contaminant predictions with human and ecological risk models, dynamic and cost-optimizing policy interventions can be customized for local contexts. This approach aims to improve human and environmental health equitably through adaptive and targeted safeguards. In summary, the synergistic integration of spatial analytics, real-time AI, and systems modeling is envisaged to democratize, discover, and decide, thereby securing water futures effectively.

## Supporting Information Available

Additional information on SERS band assignments for bacteria, polystyrene, nitrate, and PFAS (PDF)
