# Supplementary material for: Machine Learning-Assisted Surface-Enhanced Raman Spectroscopy Detection for Environmental Applications: A Review
Source: Environ Sci Technol. 2024 Nov 13;58(47):20830–48. doi: 10.1021/acs.est.4c06737 (PMC11603787; doi:10.1021/acs.est.4c06737)
Supplement: Supplementary file 1 — es4c06737_si_001.pdf [file es4c06737_si_001.pdf]

Supporting Information for

Machine Learning-Assisted Surface-Enhanced Raman Spectroscopy Detection for Environmental Applications: A Review

Sonali Srivastava[†‡], *Wei Wang*[†‡], *Wei Zhou*[§], *Ming Jin*[§] *and Peter J. Vikesland**[†‡]

[†] Department of Civil and Environmental Engineering, Virginia Tech, Blacksburg, Virginia 24061, United States

[‡] Virginia Tech Institute of Critical Technology and Applied Science (ICTAS) Sustainable Nanotechnology Center (VTSuN), Blacksburg, Virginia 24061, United States

[§] Department of Electrical and Computer Engineering, Virginia Tech, Blacksburg, Virginia 24061, United States

* Corresponding Author: Peter J. Vikesland; E-mail: vikesland@vt.edu

The Supporting Information contains 1 table.

Table S1: Band assignments for the peaks identified in the SERS spectra of bacteria, microplastics, nitrate, and PFAS.

Pollutants	Functional groups	Raman bands (cm^{-1})
Bacteria	adenine ring vibration	735, 1326[35]
	C-N stretching	800, 965[36]
	amide II vibration	1556, 1633[37]
	guanine ring vibration	656[38]
	C-H bend, C=C stretching	1560[39]
	C-S stretch	655[40]
	amide III vibrations	950-1300[40]
	amide I vibrations	1660[41]
	amine/ phosphate / FAD (flavin adenine dinucleotide)	1055[42]
	polysaccharide (bacterial cell wall)	965[43]
Microplastics (polystyrene)	ring breathing vibration	1004[44]
	C-H in-plane deformation	1032[45]
	C-C stretches	1150-1200[44,45]
	ring skeleton stretches	1605[45]
Anion (nitrate)	N-O bending	719[46]
	symmetric stretching	1047[46]-1056[47]
PFAS	asymmetric stretching mode of CF_2	1300[33]
	symmetric stretching mode of CF_2	732-747[48]
	SO_3^{2-} stretching mode	1045, 1140[49]
	C-F stretching mode	1350[48]
